# Metabolic availability of amino acids in humans

**DOI:** 10.3389/fnut.2024.1400676

**Published:** 2024-05-21

**Authors:** Alyssa Paoletti, Rajavel Elango, Glenda Courtney-Martin

**Affiliations:** ^1^Research Institute, Hospital for Sick Children, Toronto, ON, Canada; ^2^Department of Pediatrics, University of British Columbia, Vancouver, BC, Canada; ^3^School of Population and Public Health, University of British Columbia, Vancouver, BC, Canada; ^4^BC Children’s Hospital Research Institute, BC Children’s Hospital, Vancouver, BC, Canada; ^5^Department of Nutritional Sciences, University of Toronto, Toronto, ON, Canada; ^6^Department of Kinesiology, University of Toronto, Toronto, ON, Canada

**Keywords:** protein quality, metabolic availability, amino acids, indicator amino acid oxidation, slope ratio assay

## Abstract

Knowledge of amino acid bioavailability and the effect of combining complementary protein sources are required to determine how to best meet an individual’s protein and indispensable amino acid needs. Traditionally, protein quality of foods has been assessed using digestibility data. Digestibility may overestimate bioavailability of some amino acids particularly those more susceptible to heat and processing. The indicator amino acid oxidation (IAAO) method has been validated and applied to determine amino acid bioavailability termed metabolic availability of the first limiting amino acid of a proteinaceous food. The metabolic availability of the limiting amino acid in the test protein is determined as a ratio of the indicator amino acid oxidation response to graded intakes of the test protein compared to the indicator response to a reference protein (crystalline amino acid patterned after egg protein). The IAAO method has also been applied to assess the effect of protein complementation directly in humans on the overall protein quality of the diet. The results demonstrate that protein complementation augments the limiting amino acid supply and increases protein synthesis.

## 1 Introduction

The nutritive value of proteins depends on their amino acid content and their bioavailability (1). Therefore, to understand the extent to which dietary proteins can meet the requirements for indispensable amino acids, the requirement for each indispensable amino acid and their bioavailability in food proteins must be known at each stage of the life cycle. Digestibility and bioavailability are often used interchangeably, based on the assumption that if an amino acid is digested and absorbed, it is available to the body for protein synthesis. *Digestibility* refers to the net absorption of amino acid across the intestine whereas *bioavailability* refers to the proportion of the total amino acids that are digested and absorbed in a form suitable for protein synthesis (2). Using growth response and amino acid retention in growing pigs Batterham et al. demonstrated that bioavailability was lower than ileal digestibility for lysine, threonine, methionine and tryptophan (2) due to their increased sensitivity to heat and processing. In the quantity normally consumed, plant protein sources may fail to fulfil the requirements for these indispensable amino acid which makes them the *limiting amino acid* in plant-based diets (3). For those consuming a strict plant-based diet, the limiting amino acid will restrict the body’s capacity to build proteins (4). Therefore, the limiting amino acid and its bioavailability are primary determinants of a food’s protein quality.

The provision of complementary protein sources each containing a limiting amino acid in the same meal, helps to increase the content of that amino acid in the diet. This is known as *protein complementation*, a traditional and practical strategy used to improve the protein quality of plant-based diets (4). Thus, an ideal tool for protein quality assessment should possess the capacity to evaluate the effectiveness of complementation so that recommendations can be made regarding appropriate ratios required to optimize protein quality (5) of the diet. This review will discuss the application of the IAAO method in the determination of protein quality of foods for human consumption with a focus on its novel application to assess the effects of protein complementation directly in humans.

## 2 Evaluation of protein quality using the slope ratio-IAAO method

**Figure 1:**
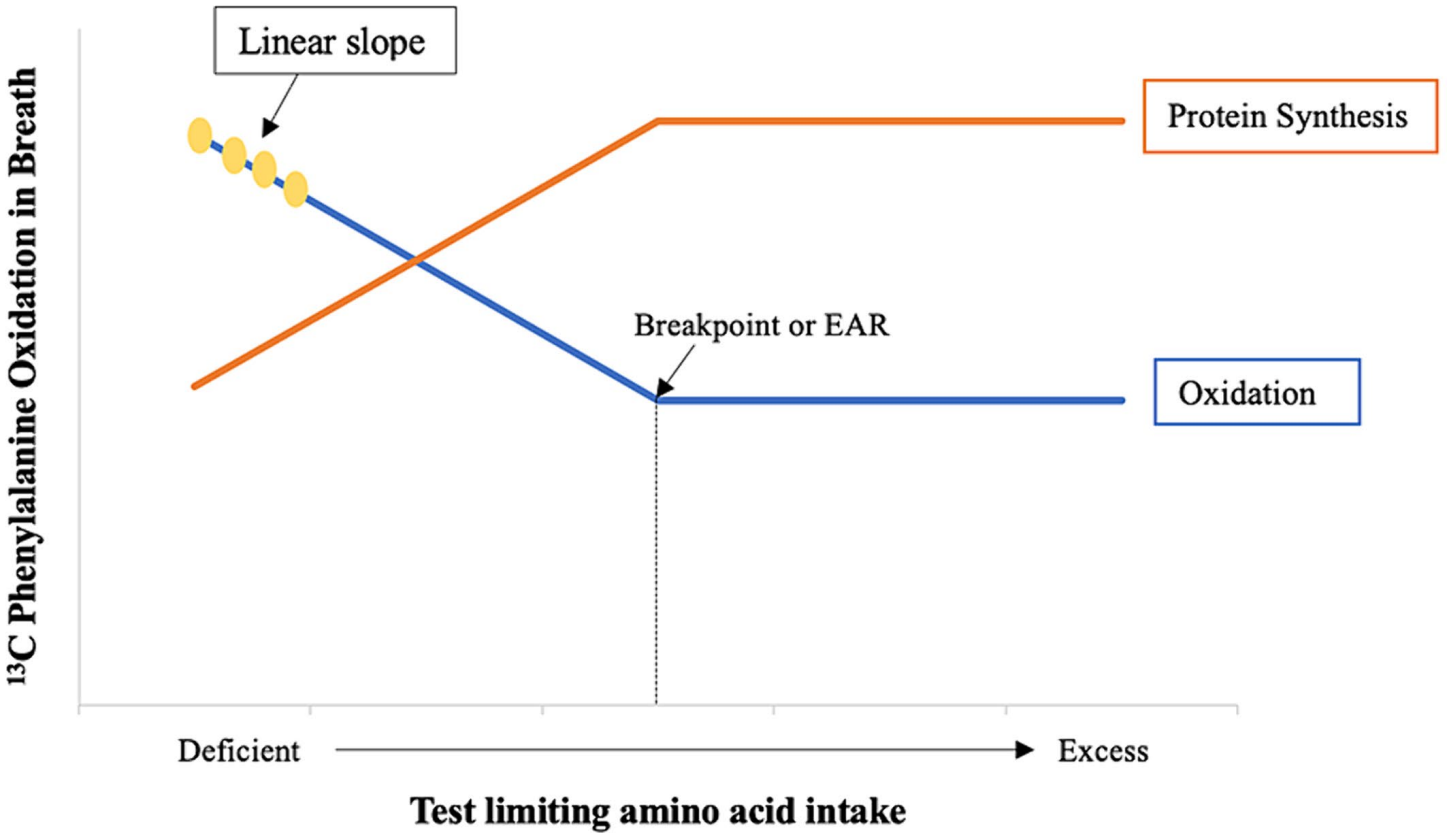
The inverse relationship between protein synthesis and oxidation (8). Adapted from Levesque et al. (9).

The IAAO method has been applied to the study of protein quality of foods for human consumption by measuring the bioavailability termed metabolic availability (6, 7) of the first limiting amino acid in foods. The food under study is first analyzed for its protein and amino acid composition to identify the first limiting amino acid. Below the limiting amino acid requirement, the decrease in IAAO will be linear with increasing intakes of the limiting amino acid (Figure 1). This patterned IAAO response can be used to measure metabolic availability of a proteinaceous food containing a limiting amino acid because decreased oxidation of the indicator amino acid is inversely related to protein synthesis (5, 8). In order to do so, the IAAO response is combined with *slope-ratio* principles, which can be considered the gold standard against which other bioavailability methods are judged (2).

Thus, the IAAO response to intakes of the test limiting amino acid from a food (protein-bound amino acid) is measured and compared to the IAAO response to the same intakes of the test limiting amino acid from a reference protein. The reference protein is provided as free, crystalline amino acid since it has been shown that the true digestibility of crystalline amino acids in pigs is essentially 100% (10). Therefore, the relative ratio of the IAAO response in the food compared to the IAAO response in the reference protein is proportional to whole-body bioavailability of the limiting amino acid (5–7). It captures both absorption, and availability of the amino acid for metabolic processes (i.e., protein synthesis). Hence, any dietary losses of amino acids during these biological processes are accounted for (5–7).

### 2.1 Validating the application of the IAAO method for protein quality evaluation

The application of the IAAO method to the study of protein quality was first validated in piglets (6) in which the bioavailability of lysine from raw and heated peas were assessed. The test diets (raw and heated peas) supplied lysine below its requirement (11) to ensure that the IAAO response was linear with increasing intakes of dietary lysine. The IAAO response to feeding the raw peas and heated peas were compared to the IAAO response to feeding free lysine (reference diet) separately to estimate the metabolic availability of lysine from each diet. Free lysine was then added back to the heated peas to assess whether the method was sensitive to the effects of lysine supplementation. The bioavailability of lysine from raw and heated peas were determined to be 88 and 55%, respectively (6). These estimates were comparable to estimates obtained by Van Barneveld and colleagues using a slope-ratio growth assay (12). When free lysine was added back to the heated peas, a decline in the IAAO response was observed demonstrating increased metabolic availability of lysine for protein synthesis (6). This was similarly demonstrated by Van Barneveld and colleagues in which the addition of free lysine to heated peas reversed the growth depression in piglets caused by the heated peas (12). Overall, the distinct IAAO response to either raw peas, heated peas, or heated peas plus free lysine demonstrates that phenylalanine oxidation directly reflects the bioavailability of lysine and that the changes in oxidation were due to changes in metabolically available dietary lysine for protein synthesis. It also highlights the ability of the method to capture the effects of heat processing which is important to human nutrition in which many foods are processed (i.e., subject to the effects of heat or chemical treatment) before consumption.

### 2.2 Criteria required for measuring protein quality using the IAAO method

To quantify the bioavailability of a limiting amino acid in a food, important criteria must be met. First, the test amino acid must be the first limiting amino acid responsible for driving the changes in the IAAO response (5) and must be the only limiting amino acid in the diet. This is achieved by providing a diet patterned after the amino acid composition of egg protein. Thus, all amino acids are fed in excess of their requirements except for the test amino acid. The indicator amino acid (i.e., most commonly phenylalanine in the presence of excess tyrosine) is also provided above its requirement and is held constant throughout all diets to ensure indicator oxidation is due only to changes in the intakes of the test amino acid. The dispensable amino acid alanine is used to balance the nitrogen content of the diets as the intakes of the test amino acid is varied (5). For protein quality studies, the indicator amino acid is provided at intakes around 30 mg · kg^−1^ · d^−1^ of ^13^C-phenylalanine in the presence of excess tyrosine: 40 mg · kg^−1^ · d^−1^ (5).

The IAAO response to changes in the dietary intake of the test amino acid (in mg · kg^−1^ · d^−1^) must be linear (statistical validity) to permit the calculation of bioavailability according to the principles of the slope-ratio assay (13). This is accomplished by providing intakes of the limiting amino acid that fall below the lower confidence limit of its dietary requirement (6, 14–16). Therefore, it is necessary to know the dietary requirement of the test amino acid determined by the IAAO method *a priori* to appropriately select intakes located on the linear portion of the IAAO slope (Figure 1). Additionally, the bioavailability of the limiting amino acid in the food is measured relative to a reference protein which is fed as free crystalline amino acids. This reference protein is assumed to be 100% bioavailable (10) which means that the IAAO reference slope signifies the maximal unit increase in protein synthesis (5).

To compare the IAAO response of the test protein to the IAAO response of the reference protein, the slopes require a common intercept (fundamental validity) (13). Thus, the design of the study diets must be carefully considered. For example, it is possible to test 0 mg · kg^−1^ · d^−1^of the limiting amino acid from the reference diet (free crystalline amino acid) and observe a biologically meaningful IAAO response. However, since amino acid containing foods are the test source of the limiting amino acid, it is not possible to provide 0 mg · kg^−1^ · d^−1^ of the limiting amino acid from the test food. Therefore, the IAAO response from the reference diet at 0 mg · kg^−1^ · d^−1^ could not be compared to the IAAO response to feeding 0 mg · kg^−1^ · d^−1^ from the test food. To reconcile this statistical issue, a base amount of the crystalline form of the limiting amino acid is added to all the diets, both reference and test. This basal amount of the test amino acid supplied across all intakes (both reference and test diets) assumes that the IAAO response is the same at this basal intake which enables the two regression lines (reference and test) to intersect at a common intercept (13).

The IAAO response must demonstrate good repeatability to permit accurate quantification of metabolic availability. This is achieved with a repeated measures design which reduces intra individual variation. The repeated measures design is made possible by the use of the minimally invasive IAAO protocol which combines a short 2 day adaptation (17) period to a 9 h fed-state oxidation day (14–16, 18, 19). Despite this, a major drawback of the IAAO method is that only one amino acid is evaluated at a time, rendering the procedure time consuming and expensive compared to the other available digestibility-based methods. However, dietary intakes are tightly controlled, thus, low variation (i.e., CV = <10%) in the IAAO derived bioavailability estimates have been obtained (14, 18, 19). The IAAO combined slope-ratio method has been successfully applied in humans to assess the protein quality of a variety of commonly consumed grains and pulses (14, 18, 19). Most recently, IAAO method has been adapted and applied to study the effectiveness of protein complementation directly in humans.

## 3 Novel application of IAAO to assess protein complementation in humans

Knowledge of the limiting amino acid content and its bioavailability from plant proteins determined using the IAAO combined slope-ratio method permits the direct quantification of the individual amounts of each individual plant foods required to fulfil an individual’s protein and amino acid needs. In this way, the premise on which protein complementation is based is met by ensuring adequate quantities of all amino acids are present at the site of protein synthesis (3). Thus, in the context of a meal, the goal is to augment the limiting amino acid intake by combining two complementary plant proteins; one limiting and the other sufficient in that amino acid. For example, pulses like chickpeas are limiting in methionine (20) whereas rice contains sufficient methionine. To effectively complement a chickpea-based diet with a grain such as rice, knowledge on the bioavailability of methionine in both protein sources is required.

### 3.1 Rice and chickpea mixed meal

The IAAO method was first applied to the study of protein complementation in humans, by combining chickpeas and rice in a mixed meal (14). In that study, the separately determined bioavailability of methionine from steamed rice (bioavailability = 100%) and cooked chickpeas (bioavailability = 63%) were used to design a mixed-meal of cooked rice plus chickpea (18). The aim was to enhance the methionine intake from chickpea protein by adding methionine from rice protein. The impact of complementation was assessed by comparing the IAAO responses at each methionine intake from the cooked chickpeas alone to that of the IAAO responses at the same methionine intakes from the cooked chickpea plus rice after adjusting for the bioavailability of methionine in chickpeas. When this was done, a decline in IAAO (6–14%) at each methionine intake was observed compared to the IAAO responses of methionine intakes from the cooked chickpeas alone (18). This demonstrates an increase in net protein synthesis due to the increased intake of methionine from the complementation of the two protein sources. Thus, more methionine is provided in the mixed diet that is available from chickpea alone reflecting superior protein quality of the mixed meal. The effect of protein complementation by combining millet (limiting in lysine but sufficient in methionine) with lentils (sufficient in lysine but limiting in methionine) have also been assessed (19).

The metabolic availability values obtained using the IAAO method and the results of the protein complementation studies are outlined in Table 1.

**Table 1:** Metabolic availability values and the effect of protein complementation derived using the IAAO method in humans.

Food	Amino acid	Population	Metabolic availability (%)	Complementary food	% Decrease in indicator oxidation	Reference
Soy protein isolate	Methionine	Young adult males	71.8			(21)
Casein	Methionine	Young adult males	87.4			(21)
Cooked white rice	Lysine	Young adult males	97			(14)
Oven browned white rice	Lysine	Young adult males	70			(14)
Cornmeal	Lysine	Young adult males	71			(15)
Cornmeal	Tryptophan	Young adult males	80			(15)
Millet	Lysine	Young adult males	97	Lentils	27	(19)
Lentils	Lysine	Young adult males	80			(19)
Sorghum	Lysine	Young adult males	94	Lentils	19	(16)
Chickpeas	Methionine	Young adult males	63	Rice	14	(18)
Rice	Methionine	Young adult males	100			(18)
Lentils	Methionine	Young adult males	69	Rice	16	(22)
Warm rice	Lysine	School-aged children	97.5			(23)

## 4 Conclusion

The IAAO method was validated in pigs and successfully applied in humans to determine the metabolic availability of the limiting amino acid in plant foods and to assess the effect of protein complementation. Nonetheless, the IAAO method has some limitations. As mentioned above, only one indispensable amino acid is evaluated at a time, rendering the procedure time consuming and expensive to study several foods. Another limitation concerns protein sources with more than one limiting amino acid in which case the bioavailability of each limiting amino acid will need to be determined separately. However, the method is non-invasive which makes it suitable for application in vulnerable populations like children, pregnant women and older adults. A key advantage is IAAO measures metabolic availability rather than digestibility alone, and hence can be useful for assessing the effect of processing and in developing reference values for major food protein sources and in the validation of new techniques for estimating amino acid bioavailability.

## Author contributions

AP: Writing – original draft. RE: Writing – review & editing. GC-M: Writing – review & editing, Conceptualization, Funding acquisition, Project administration, Resources, Supervision.
